# Reference invasive tests of microvascular injury in myocardial infarction

**DOI:** 10.1136/heartjnl-2017-311695

**Published:** 2017-08-05

**Authors:** Annette Marie Maznyczka, Peter McCartney, Colin Berry

**Affiliations:** 1 British Heart Foundation Glasgow Cardiovascular Research Centre, Institute of Cardiovascular and Medical Sciences, University of Glasgow, Glasgow, UK; 2 West of Scotland Heart and Lung Centre, Golden Jubilee National Hospital, Glasgow, UK

**Keywords:** Cardiac Catheterization And Angiography, Cardiac Magnetic Resonance (cmr) Imaging, Acute Myocardial Infarction, Percutaneous Coronary Intervention

In patients with ST-segment elevation myocardial infarction (STEMI), primary percutaneous coronary intervention (PCI) successfully restores normal antegrade flow in the infarct-related artery in nearly 99% of patients. However, approximately half of all STEMI patients have failed microcirculatory reperfusion, as reflected by microvascular obstruction (MVO), and one-third have myocardial haemorrhage, reflecting severe, ‘downstream’, potentially irreversible, microvascular injury.^1^

MVO is the ‘Achilles Heel’ of primary PCI, yet clinicians are generally unaware of the occurrence of MVO and myocardial haemorrhage in their patients, unless cardiac magnetic resonance (CMR) is performed. However, CMR is not done routinely. Other established investigations for detecting failure of myocardial reperfusion, such as angiographic, or electrocardiographic parameters, lack sensitivity and reproducibility in clinical practice.

Immediate invasive measurement of microvascular resistance at the time of PCI has the potential to optimise the approach to therapeutic interventions by: (1) acutely identifying patients at high risk of MVO who are most likely to benefit from adjunct therapy, for example, with glycoprotein IIb/IIIa inhibitors, (2) targeting novel therapies in clinical trials to patients with evidence of microvascular dysfunction and (3) allowing immediate evaluation of the efficacy of reperfusion therapy. However, invasive tests of the efficacy of myocardial reperfusion in STEMI patients have been hampered by a number of methodological and technical shortcomings. The ideal acute invasive test of microvascular perfusion and dysfunction should be reliable and reproducible, operator independent and easy to perform with standard PCI equipment.

**Figure 1:**
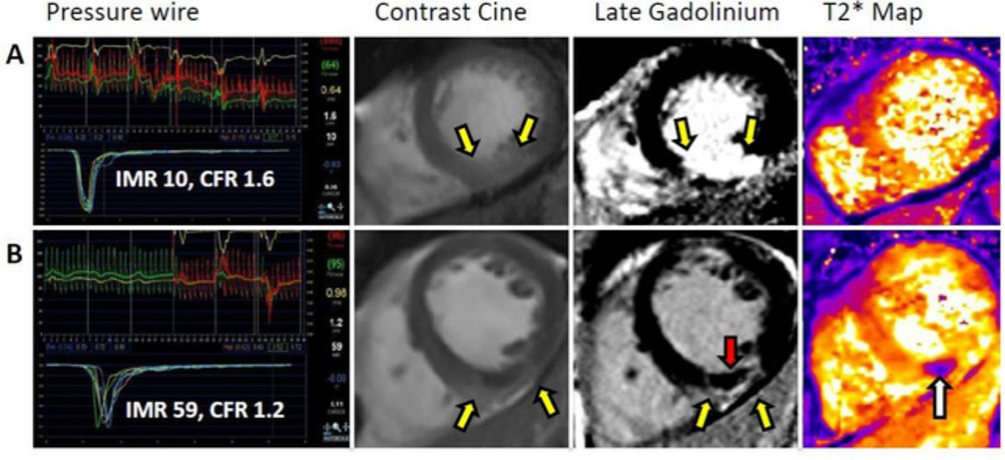
Two patients, both with acute inferior ST-segment-elevation myocardial infarction treated successfullywith primary percutaneous coronary intervention (PCI). Each patient had thrombolysis in myocardial infarction (TIMI) grade 3 flow at the end of PCI. Cardiac magnetic resonance (CMR) imaging was performed at 3 days postreperfusion in both patients. (A) Patient with normal index of microcirculatory resistance (IMR), low coronary flow reserve (CFR), an inferior infarct but no microvascular obstruction (MVO) or myocardial haemorrhage on CMR 2 days later. The diagnostic guide wire study of culprit artery microvascular function at the end of primary PCI indicated an abnormal CFR (1.6) but a preserved IMR (10). Late gadolinium contrast-enhanced CMR revealed an inferior infarct with no evidence of MVO (middle image, yellow arrows). (B) A patient with high IMR, low CFR and haemorrhagic infarction on CMR. The diagnostic guide wire study of culprit microvascular function immediately after primary PCI indicated severe microcirculatory dysfunction (IMR 59 and CFR 1.2). T2*-CMR (far right image) revealed myocardial haemorrhage (white arrow) within the infarct core. Contrast-enhanced CMR revealed MVO (middle image, red arrow) within the bright area of infarction. The MVO within the infarct core spatially corresponded with the myocardial haemorrhage.

Currently, the index of microvascular resistance (IMR) has the most extensive evidence base to support its use as a reference test of culprit artery microvascular function in patients with acute STEMI. IMR is a thermodilution-derived index, measured using a guide wire that combines a pressure and temperature sensor. Specifically, IMR is defined as distal coronary pressure multiplied by the mean transit time of a 3 mL bolus of saline at room temperature during maximal coronary hyperaemia. IMR measured at the end of primary PCI reproducibly reflects the extent of MVO, observed on CMR. Moreover, an IMR >40 (postprimary PCI) reliably predicts mortality and heart failure (independent of infarct size) at 1 year.^2 3^ Importantly, IMR derived at peak hyperaemia has less haemodynamic dependence than coronary flow reserve (CFR), providing a more reproducible assessment of the microcirculation in patients with STEMI (figure 1).

CFR reflects epicardial and microvascular vasodilator capacity (unlike IMR, which measures microvascular resistance); however, CFR does not allow discrimination between the two components. In a previous study, Carrick *et al* showed that IMR >40 and CFR ≤2 combined did not confer incremental prognostic value in STEMI patients.^3^ IMR was more closely associated with myocardial haemorrhage, whereas CFR (not IMR) was discriminative in patients with less severe (potentially reversible) MVO.^4^

In their *Heart* manuscript, de Waard *et al* 5 present their findings on hyperaemic microvascular resistance (HMR), compared with CFR, measured immediately after PCI for MI (n=176), as a predictor of clinical outcome and MVO (referred to as microvascular injury in their paper). HMR may be considered analogous to IMR, as they are both measures of microvascular resistance. However, unlike IMR, HMR is derived from pressure Doppler flow guide wires, thus negating the need for manual injection of saline. Specifically, HMR is the ratio between hyperaemic mean distal pressure and hyperaemic average Doppler flow peak velocity. A reliable Doppler flow velocity tracing is required for measurement; however, Doppler flow velocity signals may be inconsistent and are particularly influenced by the wire tip position. Indeed, in the current study, 8% of invasive measurements had to be excluded, due to poor Doppler flow signals, or iatrogenic coronary dissection in one patient.^5^ Compared with IMR, Doppler guide wire measurements have a steeper learning curve, the measurements are technically challenging, the amount of time involved to acquire the data may be longer and in general the Doppler wire is more expensive than the wire that is used to measure IMR. For all of these reasons, HMR is currently a method used in research protocols rather than in routine practice.

IMR also has drawbacks. A potential limitation of using IMR to assess MVO is that (unlike HMR) the location of the sensor influences transit time so should be constant during repeated measurements and in general standardised to 6–9 cm from the guide catheter. IMR has a weak association with LV mass (unlike CFR).^4^ Thus, intervessel variability in IMR measurement may exist, and the territorial extent of coronary microcirculatory disruption could be a potential confounding variable. Theoretically, for measured IMR to reflect, true microvascular resistance collateral flow needs to be taken into account, by correcting for wedge pressure, which is measured by balloon occlusion of the coronary artery. However, in the context of acute STEMI uncorrected-measured IMR correlates strongly with wedge-corrected IMR.^6^ The explanation for why collateral arteries can potentially complicate the measurement of microvascular resistance (ie, IMR or HMR) is that flow arising from collateral arteries distal to the sensor generates pressure that is transmitted through the coronary artery, hence overestimation of the distal pressure measurement and microvascular resistance.

De Waard *et al* 5 found that CFR <1.5 was predictive for the composite endpoint (death and hospitalisation for heart failure), HR: 3.5, 95% CI 1.1 to 10.8, but not for the separate components of death and heart failure hospitalisations. HMR ≥3.0 mm Hg·cm^-1^-s was more strongly predictive for the composite endpoint, HR: 7.0, 95% CI 1.5 to 33.7, was an independent predictor for its individual components and remained independently associated with the composite outcome after adjustment for baseline and procedural characteristics. Furthermore, compared with CFR, HMR was a superior predictor of MVO (defined by CMR). The authors therefore concluded that HMR is superior to CFR, for identifying MI patients with MVO who are at high risk of adverse clinical outcome.

Like IMR, HMR represents an emerging approach for the immediate assessment for MVO in the catheter laboratory, but given the considerations around its use, it may be most useful for research purposes rather than unselected real-world practice. Alternatively, IMR would seem better disposed for use in real-world practice. The findings of de Waard *et al* 5 complement those of previously published studies^3 4^ in a number of important ways. First, a broader range of MI types were included (ie, both STEMI (n=130) and non-STEMI (n=46) patients were analysed) with stable patients (without coronary artery disease) serving as a reference cohort. Second, it was a multicentre study. Third, microvascular resistance was measured using Doppler, instead of thermodilution.

The limitations of the current study^5^ need to be borne in mind. Importantly, the sample size was modest, raising the possibility of type 1 error, consecutive patients were not recruited and nearly 1 in 10 recordings were ruled out due to issues with data acquisition.

Further work in this area is warranted. In particular, the findings of the current study^5^ should be further evaluated in larger cohorts of patients. Several invasive measures of the microcirculation have been described in STEMI cohorts, including IMR,^4^ HMR,^5^ zero-flow pressure^7^ and absolute microvascular resistance (which in contrast to IMR requires estimation of myocardial mass).^8^ Each of these indices has pros and cons. To date, only IMR, as a measure of infarct pathology and predictor of mortality, has been validated in large cohorts, and IMR is comparatively straightforward to measure. These tests of microvascular injury have potential to enable a ‘stratified medicine’ approach, in which patients identified to be at higher risk of adverse outcome may be stratified for more intensive therapy. This idea is being prospectively evaluated in T-TIME (NCT02257294), which is a phase 2, randomised, placebo-controlled clinical trial of reduced doses of intracoronary alteplase in selected higher risk patients with STEMI.

IMR and HMR have potential as novel indices for patient selection and as biomarkers of the efficacy of therapy; however, more research is needed to assess whether IMR and HMR are modifiable. Further research is also warranted to validate IMR and HMR as novel tests of the efficacy of intracoronary therapies; should this be the case, the results would support the use of IMR and HMR as a biomarker of the efficacy of therapeutic interventions designed to improve myocardial reperfusion, as assessed in future trials.

In conclusion, the study by de Waard *et al* 5 extends the evidence on the pathophysiological and prognostic importance of microvascular dysfunction in the culprit artery at the end of PCI. De Waard *et al* 5 provide original data on HMR, which adds to previous investigations of IMR and CFR, in patients with STEMI. Studies in larger cohorts are needed to explore further the utility of IMR and HMR as a therapeutic target during primary PCI and to identify and stratify higher risk patients for more intensive management.
